# Oxytocin-Motivated Ally Selection is Moderated by Fetal Testosterone Exposure and Empathic Concern

**DOI:** 10.3389/fnins.2013.00001

**Published:** 2013-01-30

**Authors:** Mariska E. Kret, Carsten K. W. De Dreu

**Affiliations:** ^1^Department of Psychology, University of AmsterdamAmsterdam, Netherlands

**Keywords:** oxytocin, testosterone, threat, empathy, social decisions

## Abstract

In humans, the hypothalamic neuropeptide oxytocin shifts the individual’s focus on self-interest toward group-serving cognitions and decision-making. Here we examine this general tendency in the context of group formation, where individuals included into their group (or not) 18 targets morphed as having low or high-threat potential (with high-threat targets being beneficial to group-interests but potentially hurting the recruiter’s self-interest). Ninety healthy males self-administered oxytocin or placebo in a randomized double-blind, placebo-controlled study design, had their hands scanned to derive fetal testosterone vs. estradiol exposure from their 2D:4D ratio, and self-reported on their chronic empathic concern. Multilevel regression models revealed that when given oxytocin rather than placebo, individuals with low fetal testosterone priming included low-threat targets more and high-threat targets (somewhat) less. Individuals with high fetal testosterone (i.e., low estradiol) exposure, however, included high-threat targets more, and low-threat targets less when given oxytocin rather than placebo. Second, when given oxytocin rather than placebo, individuals with low empathic concern included low-threat targets more and high-threat targets less. Individuals with high empathic concern, however, included high-threat targets more, and low-threat targets less when given oxytocin rather than placebo. We conclude that oxytocin shifts the individual’s focus from self to group-serving cognition and decision-making, and that these tendencies are stronger for males with high rather than low fetal testosterone vs. estradiol exposure, and high rather than low empathic concern. Implications and avenues for future research are discussed.

## Introduction

Throughout evolution, humans have moved in and out of groups, relaxing and tightening their interdependencies with others, and selecting and rejecting others into more or less closely knit social units that function well and provide their individual members with protection and survival opportunities (Kameda et al., 2005; Bowles and Gintis, 2012). Whereas the behavioral sciences identified a host of social and personality factors underlying human socialization, bonding, and group formation and maintenance (e.g., Baumeister and Leary, 1995; Rusbult and Van Lange, 2003; Mikulincer and Shaver, 2010; Ellemers, 2012), these issues received scant attention in the biological and neurosciences. However, because bonding, socializing, and forming groups provides strong survival benefits to the individual (Darwin, 1859), such group formation tendencies may rest on evolved neurohormonal circuitries (De Dreu, 2012a). Here we examine this possibility, and study whether and how (i) possible effects of the hypothalamic neuropeptide oxytocin on group formation are moderated by (ii) the ratio of fetal testosterone vs. estradiol exposure as revealed by variation in the relative length of the second (index) to fourth finger (2D:4D ratio; Manning et al., 2002), and (iii) chronic empathic concern (Davis, 1983; Frith and Singer, 2008).

### Oxytocin motivates group-serving cognition and decision-making

In forming and expanding one’s group, humans may select group-members based on characteristics such as the other’s attractiveness, friendliness, trustworthiness, or social status. Especially in a competitive setting, humans face the dilemma between including strong, domineering others that strengthen the group and provide protection against outside threat vs. engaging submissive and trustworthy others that may promote rather than threaten the recruiter’s personal interest. Searching for, and selecting strong others with high-threat potential serves group-interests more than personal interests, whereas inviting submissive others with low-threat potential serves personal interests more than group-interests (Kurzban et al., 2001; Benenson et al., 2009). As such, coalescing with strong, domineering others may be seen as part of the general inclination to serve group rather than immediate self-interest, and this in turn may be driven by the same neurobiological circuitries as other forms of group-serving behaviors such as parochial altruism, social attachment, and parental care (Madden and Clutton-Brock, 2011; De Dreu, 2012a).

Among the possible neural circuitries underlying group-serving cognition and behavior, the oxytonergic circuitry clearly stands out as most promising. Oxytocin is an evolutionary ancient, nine-amino-acid neuropeptide produced in the hypothalamus. Functioning as hormone and neurotransmitter, it targets the amygdala, hippocampus, and regions of the spinal cord that regulate the parasympathetic branch of the autonomic nervous system (Ludwig and Leng, 2006; Neumann, 2008; Rodrigues and Sapolsky, 2009). Oxytocin interacts with the hypothalamic-pituitary-adrenal axis to attenuate stress responses, and this has a pervasive influence throughout both the body and the brain (Neumann, 2008; Heinrichs et al., 2009; Bos et al., 2012). For example, oxytocin reduces cortisol levels after exposure to stressors, inhibits cardiovascular stress responses, and modulates brain areas and neural circuitries involved in processing fear-related information (Kirsch et al., 2005; Baumgartner et al., 2008; for reviews see Heinrichs et al., 2009; Bos et al., 2012). Furthermore, at least in non-human mammals, oxytocin interacts with reward processing circuitries like the inferior frontal gyrus, the caudate nucleus, and the nucleus accumbens (Skuse et al., 2005; Ludwig and Leng, 2006; Donaldson and Young, 2008).

Oxytocin has well-established roles in reproduction and pair-bond formation (Carter et al., 2008; Donaldson and Young, 2008; Kavaliers and Choleris, 2011). In humans, it promotes social approach, trust, and cooperation (Kosfeld et al., 2005; Baumgartner et al., 2008; De Dreu et al., 2011), especially when interaction partners belong to one’s own group (De Dreu et al., 2010). Thus, individuals given oxytocin rather than placebo conform to the preferences of their own group but not to those of out-groups (Stallen et al., 2012), display more positive attitudes toward fellow group-members (De Dreu et al., 2011), cooperate more within their group (De Dreu et al., 2010; Israel et al., 2012), and display greater competition toward (out-group) rivals that threaten the members of one’s group (Shamay-Tsoory et al., 2009; De Dreu et al., 2010; Hahn-Holbrook et al., 2011; De Dreu, 2012a).

These and related studies together reveal that oxytocin plays a critical role in shifting the individual’s focus on immediate personal interest, toward a broader focus on (long-term) group-interest (De Dreu, 2012a). For group formation and newcomer selection, this implies that oxytocin motivates a preference for allies with high-threat potential more than for allies with low-threat potential (who serve the recruiter’s immediate self-interests more). Indeed, Evans et al. (2010) showed that intranasal administration of oxytocin reduced aversion of angry faces, and De Dreu (2012b) showed that, in the context of inter-group competition, individuals who inhaled oxytocin rather than placebo were more likely to select allies that had high rather than low-threat potential (i.e., were high on dominance and low on trustworthiness; Oosterhof and Todorov, 2008). Furthermore, under oxytocin rather than placebo, high-threat targets were perceived as more useful allies, and their assessment of usefulness accounted for the decision to include targets with high rather than low-threat potential. From this, it appears that at least under oxytocin, the motivation to include high-threat members is driven by the desire to protect and promote the group, more than by reduced fear of being hurt in one’s self-interests.

### Oxytocin’s effects depend on fetal testosterone exposure

Neurohormones such as oxytocin influence the nervous system at a functional level by changing the activity of a given neural circuitry, or at the structural level by changing the architecture and/or connectivity of different nodes of the neural circuit. Compared to the usually rapid and short-lived functional effects on neural excitability and neurotransmission, structural effects are slow and long-lasting and can include the recruitment and/or removal of new cells to the circuit (neurogenesis vs. apoptosis), or changes in the connectivity of the circuit (synaptic plasticity; Soares et al., 2010; Peper and Koolschijn, 2012). Structural effects come about after long-term exposure to specific triggers of hormone release, such as continued drug usage or exposure to high stress environments (Lederbogen et al., 2011), because of hormone exposure at critical phases in pre- and post-natal brain development, or some combination.

Here we conjecture that structural changes due to fetal testosterone vs. estradiol priming moderate acute effects of oxytocin. A brain which is shaped by prenatal exposure to high levels of estradiol or testosterone may be differentially receptive to oxytocin administration in adult life. Testosterone is a sex steroid hormone functioning as an oxytocin antagonist (Carter et al., 1988; Carter, 2003), and fetal testosterone exposure produces reliable structural effects on the brain and on behavior in adult life (Lombardo et al., 2012, also see Beach et al., 1982; Clark et al., 1988; Wayner et al., 1988). There is also evidence that estradiol produced during prenatal life influences brain structure and adult behavior (Hutchison, 1997; Bakker et al., 2006; Bakker and Baum, 2008). In both humans and non-human primates, a reliable biomarker of the ratio of fetal testosterone vs. estradiol exposure is the length of the second (index) finger relative to the ring finger (2D:4D), with lower 2D:4D reflecting higher fetal testosterone relative to estradiol exposure (Brown et al., 2002; Manning, 2002)^1^.

In humans, higher fetal testosterone exposure (a low 2D:4D) has been associated with risk tolerance (Coates and Herbert, 2008; Campbell et al., 2010), sensation seeking (Fink et al., 2006), cooperation (Millet and Dewitte, 2006), and success in team sports (Manning and Taylor, 2001, for a review see Knickmeyer et al., 2011), all characteristics of social dominance (Eisenegger et al., 2011). From these and related findings it appears that rather than driving self-interested cognition and decision-making, high fetal testosterone exposure associates with more pro-active, goal-oriented behavior, and therefore, higher fetal testosterone exposure may be associated both with more self-interested decision-making or with more group-serving decision-making (also see van Honk et al., 2011, 2012). Whether the focus is on self-interest or group-interests then depends on external factors that determine the relative salience of group-relative to self-interest. Because the availability of brain oxytocin enables a shift in focus from self-interests to group-interests, acute effects of oxytocin will be moderated by fetal testosterone exposure. Applied to group formation and ally selection, we should then find that individuals with high fetal testosterone exposure prefer group-members that have high-threat potential more when they received oxytocin rather than placebo (Hypothesis 1A); and group-members that have low-threat potential less when they received oxytocin rather than placebo (Hypothesis 1B).

### Oxytocin’s effects depend on chronic empathic concern

In forming groups and alliances, people may not only be driven by instrumental concerns regarding their self- or group’s interests, but also by more or less chronic tendencies to empathize with others (Batson, 1998; Frith and Singer, 2008). Individuals with chronic empathic concern may be more likely to affiliate and bond with others, and experience other’s imagined pain and negative emotional states more vividly, than those with lower levels of empathic concern (Davis, 1983; Singer et al., 2004). Compared to individuals with low empathic concern, those with high empathy may thus be more likely to include rather than exclude others into their group (Davis, 1996). Furthermore, individuals with high empathic concern may be more likely to focus on group-interests and forego or even sacrifice immediate self-interests – those with empathic concern may thus be more likely than individuals with low empathic concern to include others with high-threat potential and, perhaps, less likely to include others with low-threat potential.

These lines of evidence on empathic concern, combined with our conjecture that oxytocin shifts the focus from self-interest to group-interest, imply that acute effects of oxytocin on group formation and ally selection are contingent upon chronic individual differences in empathic concern. Accordingly, we predicted that especially individuals with high empathic concern prefer group-members with high-threat potential more when they receive oxytocin rather than placebo (Hypothesis 2A); and prefer group-members with low-threat potential less when they receive oxytocin rather than placebo (Hypothesis 2B).

### Summary and overview of the current study

Male subjects filled out a short questionnaire to assess their chronic empathic concern, had their right-hand scanned to infer fetal testosterone exposure from their 2D:4D ratio, and self-administered oxytocin or placebo (double-blind, randomized between-subjects design). Subjects were shown a series of targets, with facial features being morphed into neutral, low-threat, or high-threat, and for each target, subjects indicated whether they would include the target into their group. We tested predictions regarding inclusion decisions (Hypotheses 1A–2B), and explored possible effects on ratings of choice certainty, target’s perceived usefulness, and target’s perceived dangerousness.

## Materials and Methods

### Participants

Ninety males (mean age = 21.49, SD = 2.78, range 18–29) participated for €10 (approximately 13 USD). Exclusion criteria were medical or psychiatric illness, medication, smoking, and drug or alcohol abuse. The study was performed in accordance with the Declaration of Helsinki and approved by the Ethics Committee of the University of Amsterdam. All participants provided informed consent prior to the study. The data of four participants were dropped prior to hypothesis testing because they had extremely fast reaction times paired with odd response patterns such as always pressing the same button or in turn responding with “yes” and “no”). We conjectured they did not take the task seriously.

### Materials

As targets for selection we used six different actors’ faces, that were morphed into low-threat, high-threat (as in De Dreu et al., 2012), or neutral (Oosterhof and Todorov, 2008; www.facegen.com), yielding a total of 18 targets. Although people rely on a multitude of cues when perceiving and interpreting faces, Oosterhof and Todorov (2008) identified trustworthiness and dominance as the two orthogonal dimensions that are sufficient to describe face evaluation. While face-trustworthiness is more sensitive to features signaling whether the person should be avoided or approached, dominance evaluation is more sensitive to features signaling physical strength/weakness. Threatening faces should be both untrustworthy (signaling that the person may have harmful intentions) and dominant (signaling that the person is capable of causing harm). Although these computer faces are somewhat artificial, the advantage is that other features of the face (e.g., symmetry) can be kept constant, thus creating optimal conditions for a clean hypothesis test (Oosterhof and Todorov, 2008; Said et al., 2011). Furthermore, using the same low and high-threat targets as used in De Dreu (2012b) enables a near-exact replication of their findings.

### Independent variables and experimental procedures

Participants came in-groups of two to six individuals, and were seated individually in soundproof cubicles. They were randomly assigned to the oxytocin or placebo group and tested individually. Participants self-administered, under experimenter supervision, a single intranasal dose of 24 IU placebo or oxytocin (Syntocinon-Spray Novartis; three puffs per nostril, with 1 minute in between puffs). The placebo contained all the active ingredients except for the neuropeptide, and was manufactured by Stichting Apothekers Haarlemse Ziekenhuizen in coordination with the pharmacy at the Amsterdam Medical Center, adhering to the European Union guidelines on Good Manufacturing Practice and Good Clinical Practice. Placebos were delivered in the same bottles as Syntocinon.

Following Treatment, the experimenter left the cubicle and participants filled out the Dutch translation of the seven-item Empathic Concern scale (Davis, 1983; de Corte, 2007). It measures the participant’s feelings of warmth, compassion, and concern for others (always 1 = does not describe me well, to 5 = describes me very well; Cronbach’s α = 0.775; *M* = 3.07, SD = 0.48)^2^. Hereafter, participants proceeded with a series of unrelated, computer-guided tests.

Because effects of oxytocin plateau approximately 35 minutes after administration (Baumgartner et al., 2008), the computer switched to the instructions for the main task after 30 minutes. Participants were engaged in a behavioral game in which they made a number of choices between cooperation and non-cooperation (i.e., BG-Prisoner’s Dilemma; De Dreu, 2012b) and then proceeded to the current task. They read a brief introduction stating that oftentimes people form groups by selecting others or not, and that they would be shown a series of pictures of faces, each time answering whether they would include this person into their group or not. In total, participants were shown 18 pictures, each on a new screen and randomized per participant. For each picture they answered a series of questions (see “dependent variables” below).

At the end of the experiment, and before debriefing, participant’s right-hand was scanned to calculate the ratio of the length of the index finger to the length of the ring finger (2D:4D), as an indicator of fetal testosterone exposure. We collected participants’ hand scans and computed digit lengths from the crease closest to the finger to the fingertip using photo-editing software (*M* = 0.96, SD = 0.03), a method that has been previously validated by comparing it to bone measurements taken from x-rays (Manning, 2002).

### Dependent variables

For each target, participants indicated whether they would include the target in their group (0 = NO; 1 = YES), and then how certain they were of their decision, how useful, and how dangerous they judged the target (always 1 = not at all, to 5 = very much). The latter three questions were presented in random order, and the target’s picture remained visible on the computer screen.

## Results

### Statistical analyses

Hypotheses were tested using generalized linear mixed models with a binominal distribution for the (binary) inclusion decisions, and with linear distributions for the other dependent (continuous) variables. Fixed factors included Treatment (oxytocin vs. placebo; between-subjects) and Target’s Threat Potential (low vs. neutral vs. high; within-subjects), as well as their interactions with (continuous and centered) 2D:4D and Empathic Concern. A random intercept was included, as well as a random intercept for each trial. This allowed each trial to have its own intercept across participants, over and beyond the random intercept per participant. The initial models included all the predictors of interest, and to create the final model, non-significant fixed factors were deleted one by one (see Table 1). This statistical approach is advocated in Garson (2012). Fetal testosterone exposure (2D:4D) did not correlate with empathic concern (*r* = −0.022, *p* = 0.838, and statistical conclusion validity appeared not threatened by multicollinearity (Empathic Concern: Tolerance = 0.903, VIF = 1.107, λ = 1.005, Condition Index = 1.142; 2D:4D: Tolerance = 0.998, VIF = 1.002, λ = 0.685, Condition Index = 1.383).

**Table 1:** **Tests of model effects**.

	Decision		Certainty°		Usefulness°		Dangerousness°	
	*F*	Sig.	*F*	Sig.	*F*	Sig.	*F*	Sig.
Treatment	0.179	0.672	0.988	0.321	0.684	0.409	0.282	0.596
Target’s threat	0.760	0.468	0.829	0.369	0.586	0.450	41.723	**0.000**
2D:4D	0.010	0.921	3.387	0.067			8.963	**0.003**
Treatment × 2D:4D	0.006	0.939	6.023	**0.015**			7.234	**0.007**
Target’s threat × 2D:4D	6.027	**0.002**	3.695	0.055			12.865	**0.000**
Treatment × target’s threat × 2D:4D	4.064	**0.017**	6.493	**0.011**			5.795	**0.016**
Empathic concern	4.597	**0.032**			6.164	**0.013**		
Treatment × target’s threat	0.357	0.700	1.670	0.197	0.960	0.328	0.365	0.546
Treatment × empathic concern	0.009	0.926			6.655	**0.010**		
Target’s threat × empathic concern	1.904	0.149			7.767	**0.005**		
Treatment × target’s threat × EC concern	8.897	**0.000**			4.137	**0.042**		

°Included targets only

*Significant factors are printed in bold*.

### Inclusion decisions (hypotheses 1A–2B)

Hypotheses on selection decisions were tested in a generalized mixed model with selection decision (0 = no, 1 = yes) as binary dependent variable. Table 1 shows the final model results. We observed an interaction between 2D:4D ratio and Target’s Threat Potential, *F*(2, 1.530) = 6.027, *p* = 0.002, which was qualified by the predicted three-way interaction among Treatment, 2D:4D and Target’s Threat Potential, *F*(2, 1.530) = 4.064, *p* = 0.017.

**Figure 1:**
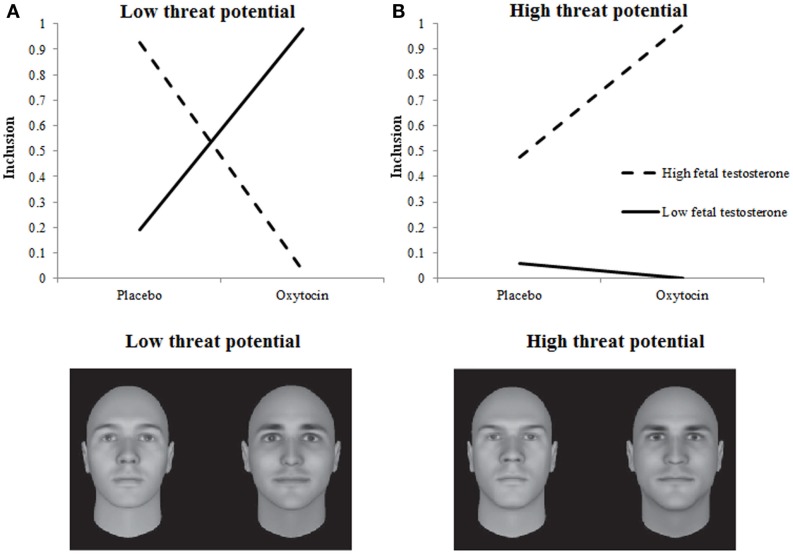
**Fetal testosterone exposure moderates effects of oxytocin on inclusion decisions**. Low-threat targets [**(A)** examples in bottom left panel] are preferred less and high-threat targets [**(B)** examples in bottom right panel] are preferred more by individuals with high fetal testosterone vs. estradiol exposure when given oxytocin rather than placebo. Fetal testosterone vs. estradiol prenatal priming ratio was included as a continuous variable in our model. For visualization purposes, we plotted the interaction with this continuous variable centered once at +1 SD (dotted lines) and once at −1 SD (solid lines).

To interpret this complex interaction, the two-way interaction among Treatment and 2D:4D is plotted separately for Target’s Threat Potential. For neutral Targets we had no *a priori* predictions but observed that neutral targets were included more by individuals with low rather than high testosterone exposure, regardless of Treatment. For high and low-threat Targets, Treatment and 2D:4D interacted, as predicted in Hypothesis 1A and 1B. When given oxytocin rather than placebo, individuals with low testosterone exposure included low-threat targets more. Individuals with high testosterone exposure, however, included high-threat targets more (Hypothesis 1A), and low-threat targets less (Hypothesis 1B) when given oxytocin rather than placebo (see also Figures 1A,B). Put differently, the pattern of inclusion of low vs. high-threat targets observed earlier in De Dreu (2012b) is replicated among individuals with high testosterone exposure (low 2D:4D ratios), and tends to reverse among individuals with low testosterone exposure (high 2D:4D ratios).

**Figure 2:**
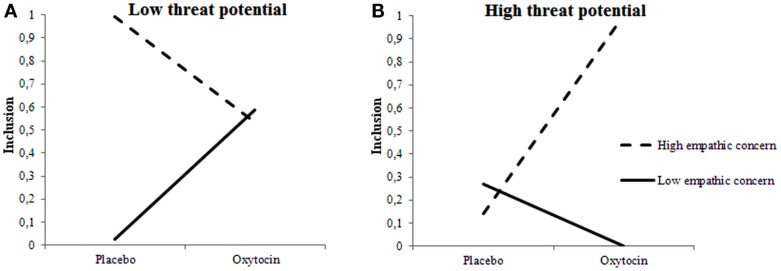
**Empathic concern moderates effects of oxytocin on inclusion decisions**. Low-threat targets **(A)** are preferred less and high-threat targets **(B)** are preferred more by individuals with high empathic concern when given oxytocin rather than placebo.

Hypotheses about the moderating influence of Empathic Concern received support too. Table 1 shows that the higher participants’ empathic concern the more often they decided to include someone into their team, *F*(1, 1.533) = 4.597, *p* = 0.032. This main effect was qualified by a three-way interaction among Treatment, Empathic Concern, and Target’s Threat Potential, *F*(2, 1.533) = 8.897, *p* = 0.0001. Again, we plotted the two-way interaction among Treatment and Empathic Concern separately for Targets with low, neutral, and high-threat potential. Neutral Targets were included more by individuals with low rather than high testosterone exposure, regardless of Treatment. For low and high-threat Targets, Treatment, and Empathic Concern interacted as predicted in Hypothesis 2A and 2B. When given oxytocin rather than placebo, individuals with low empathic concern included low-threat targets more and high-threat targets (somewhat) less. Individuals with high empathic concern, however, included high-threat targets more, and low-threat targets less when given oxytocin rather than placebo (see Figures 2A,B). Put differently, the pattern of inclusion of low vs. high-threat targets observed earlier in De Dreu et al. (2012) is replicated among individuals with high empathic concern, and tends to reverse among individuals with low empathic concern.

### Exploratory analyses

#### Choice certainty

**Figure 3:**
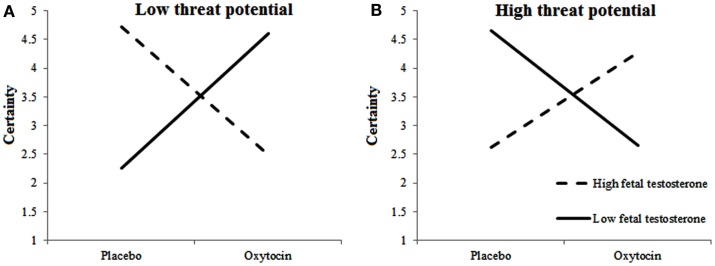
**Fetal testosterone exposure moderates effects of oxytocin on certainty of inclusion decisions**. Certainty about including low-threat targets **(A)** is lower and about high-threat targets **(B)** is higher among individuals with high fetal testosterone exposure when given oxytocin rather than placebo.

We examined the certainty of inclusion decisions, and observed a three-way interaction among Treatment, 2D:4D ratios, and Target’s Threat Potential, *F*(1, 675.096) = 6.493, *p* = 0.011 and interactions between 2D:4D ratios and Target’s Threat Potential *F*(1, 669.372) = 6.695, *p* = 0.055 and Treatment and 2D:4D ratios *F*(1, 313.070) = 6.023, *p* = 0.015. The pattern somewhat mimicked the one observed for selection decisions: When given oxytocin rather than placebo, individuals with low testosterone exposure felt more certain about included low-threat targets and less certain about included high-threat targets. Individuals with high testosterone exposure, however, felt more certain about high-threat targets, and less certain about low-threat targets when given oxytocin rather than placebo (see Figures 3A,B).

#### Target usefulness

**Figure 4:**
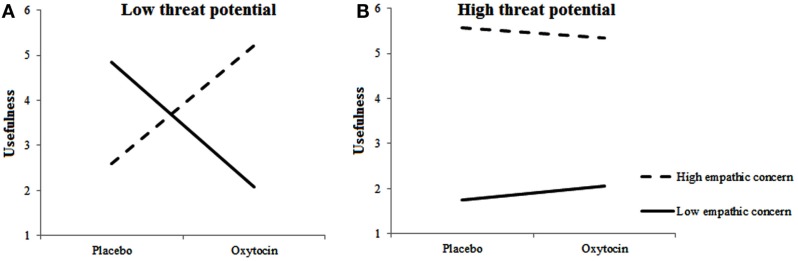
**Empathic concern moderates effects of oxytocin on usefulness of included targets**. Low-threat targets **(A)** are perceived as less useful by individuals with high fetal testosterone exposure when given oxytocin rather than placebo; High-threat targets **(B)** are perceived as more useful by individuals with high empathic concern regardless of Treatment.

For rated usefulness of included targets, we observed a main effect for Empathic Concern, *F*(1, 483.862) = 6.164, *p* = 0.013, two-way interactions between Treatment and Empathic Concern, *F*(1, 474.871) = 6.655, *p* = 0.01 and between Target’s Threat Potential and Empathic Concern, *F*(1, 680.300) = 7.767, *p* = 0.005, and a three-way interaction among Treatment, Empathic Concern, and Target’s Threat Potential, *F*(1, 691.734) = 4.137, *p* = 0.042. Figures 4A,B shows the Treatment × Empathic Concern interactions separately for high-threat and low-threat Targets. As can be seen, usefulness ratings differed as a function of Empathic Concern and Treatment mostly in the low-threat potential condition. When given oxytocin rather than placebo, individuals with high empathic concern found the low-threat targets that they included more useful. Individuals with low empathic concern, however, gave lower usefulness ratings to low-threat targets when given oxytocin rather than placebo. Highly empathic participants, regardless of treatment, rated the by them included high-threat targets as more useful than low empathic participants.

#### Target dangerousness

Dangerousness of included targets varied as a function of Target’s Threat Potential, *F*(1, 27.382) = 41.723, *p* < 0.0001, and 2D:4D, *F*(1, 362.745) = 8.963, *p* = 0.003. High-threat Targets were considered more dangerous than low-threat or neutral Targets, and individuals with low testosterone exposure perceived included targets as more dangerous than individuals with high testosterone exposure. These effects were qualified by two-way interactions between 2D:4D ratios and Target’s Threat Potential, *F*(1, 674.359) = 12.865, *p* = 0.0004, and between Treatment and 2D:4D ratios, *F*(1, 360.824) = 7.234, *p* = 0.007, as well as a three-way interaction among Treatment, 2D:4D ratios, and Target’s Threat Potential, *F*(1, 681.028) = 5.795, *p* = 0.016.

**Figure 5:**
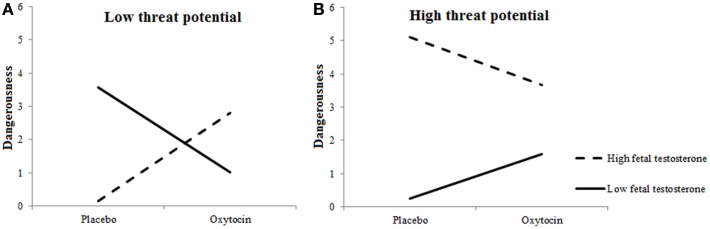
**Fetal testosterone exposure moderates effects of oxytocin on perceived dangerousness of included targets**. Low-threat targets **(A)** are seen as more dangerous and high-threat targets **(B)** as less dangerous by individuals with high fetal testosterone exposure when given oxytocin rather than placebo.

Figures 5A,B show the interactions among Treatment × 2D:4D ratios for high-threat and low-threat Targets, respectively. As can been, those with high levels of prenatal testosterone rated the by them included low-threat targets as more dangerous and high-threat targets as less dangerous under oxytocin vs. placebo. A reverse pattern was observed in those with low levels of prenatal testosterone: these participants rated the by them included low-threat targets as less dangerous and high-treat targets as more dangerous under oxytocin vs. placebo.

## Discussion

The nature and outlook of groups are shaped by continuous inclusion and exclusion decisions made by individual group-members. By including strong individuals with high-threat potential, the recruiter’s self-interests may be jeopardized yet group-interests are promoted, as adding strong individuals with high-threat potential makes the group stronger and provides protection against outside danger and rivaling out-groups. Here we observed that intranasal oxytocin (vs. placebo) motivated individuals to include high-threat targets, and to exclude low-threat targets. Importantly, these group-serving tendencies induced by oxytocin came about only in individuals with high fetal testosterone (vs. estradiol) exposure, or with high chronic empathic concern.

Results support the conjecture that a brain shaped by high fetal testosterone exposure is differentially responsive to oxytocin administration than a brain shaped by high fetal estradiol exposure (and low testosterone exposure). Although this being the very first study showing acute effects of oxytocin are moderated by fetal testosterone exposure, our findings fit with those on testosterone administration and fetal testosterone exposure reported by van Honk et al. (2011), and with the general conclusion that neurohormonal release may have both structural and acute effects that may operate independently but also interact (Soares et al., 2010; Peper and Koolschijn, 2012).

We anticipated moderation of acute oxytocin effects by proposing that higher fetal testosterone relative to estradiol exposure renders individuals more pro-active and goal-oriented, and that brain oxytocin shifts the focus from immediate self-interest to group-serving cognition and decision-making. This conjecture was further supported by exploratory analyses on choice certainty and perceived dangerousness of included targets aligned with inclusion decisions – especially individuals with high fetal testosterone exposure given oxytocin rather than placebo felt more certain about including high-threat targets, and perceived them as less dangerous. Together, this suggests that fetal testosterone exposure can be expected to moderate the effects of other variables known to shift the individual’s focus from self- to group-interest, such as team rather than individual incentives, third party instructions to cooperate rather than compete, or facing familiar and in-group protagonists rather than un-familiar individuals (e.g., Baumeister and Leary, 1995; De Dreu et al., 2008).

The finding that acute effects of oxytocin were moderated by individual differences in empathic concern fits with Bartz et al. (2011) who concluded that effects of oxytocin on social cognition and behavior depend on the individual’s chronic predispositions and personality traits. We proposed that chronic empathic concern provides a latent tendency to serve the group, which brain oxytocin turns into more manifest decision-making. This proposition was further supported by exploratory analyses on perceived usefulness of included targets – especially individuals with high empathic concern given oxytocin rather than placebo rated included high-threat targets as more useful.

Empathy is a multidimensional construct that relies on affective and cognitive component processes (Shamay-Tsoory, 2011). Our findings pertain to the *affective* empathy (empathic concern), but not to *cognitive* empathy (perspective taking, see text footnote 1). Affective and cognitive empathy relate to distinct neural circuitries. Brain regions activated by cognitive empathy include medial prefrontal regions, the superior temporal sulcus (STS), and the temporo-parietal junction (Farrow et al., 2001; Gallagher and Frith, 2003; Shamay-Tsoory et al., 2003, 2005a,b; Decety and Jackson, 2004). In contrast, brain regions activated by affective empathy mostly include somatosensory and insular cortices as well as limbic areas and the anterior cingulate cortex (Nummenmaa et al., 2008; Lang et al., 2011; for a review, see Hein and Singer, 2008). Interestingly, brain regions involved in affective empathy are more easily influenced by oxytocin than brain regions involved in cognitive empathy (Shamay-Tsoory et al., 2010), and this may explain why chronic empathic concern did and perspective taking did not moderate acute effects of oxytocin on group formation and ally selection.

We included male participants only, and cannot exclude the possibility that females respond differently to target selection when given oxytocin rather than placebo. Intranasal oxytocin sensitizes males to competitive interactions, and females to affiliation (Fischer-Shofty et al., 2012), and there is some evidence that while oxytocin down-regulates fear-responding in males, it actually boosts fear-responding in females (Lischke et al., 2012). Especially when it comes to moderation by fetal exposure to sex hormones, it may be that our findings are limited to males and new research is needed to address this possibility. Second, our target selection task did not enable individuals to compose groups. It stands to reason that well-functioning groups contain mixtures of different personality types – overrepresentation of strong, domineering individuals may be as problematic as overrepresentation of submissive individuals. The current target selection task does not inform us about the way groups are composed, and new research is needed to examine whether oxytocin, alone or in conjunction with fetal exposure to sex hormones and/or empathic concern, leads to specific preferences for group compositions. Third, we propose that those with high levels of testosterone exposure selected high-threat allies because oxytocin made them more group-focused. Although we cannot rule out that oxytocin made participants select group-members who are more like them (dominant in appearance), dangerousness ratings suggest this to be unlikely. Those with low 2D:4Ds under oxytocin rated low-threat targets as more dangerous, presumably because they would not be able to strengthen the group and not because they are unlike themselves.

Brain oxytocin enables individuals to consider group rather than self-interests (De Dreu, 2012a), and this may motivate them to include strong, domineering newcomers with high-threat potential (De Dreu, 2012b). Earlier work explicitly positioned group formation in the context of an inter-group competition. Absent such explicit reference to inter-group rivalry, we observed here that ally selection induced by oxytocin administration is highly contingent upon chronic differences in empathic concern, and prenatal testosterone vs. estradiol exposure. These findings suggest that especially among individuals set to pro-actively serve group-interests, oxytocin induced group-serving cognition and decision-making tendencies that would favor the group as a whole.

## Author Contribution

Mariska E. Kret and Carsten K. W. De Dreu designed the study. Mariska E. Kret analyzed the data. Mariska E. Kret and Carsten K. W. De Dreu wrote the paper.

## Conflict of Interest Statement

The authors declare that the research was conducted in the absence of any commercial or financial relationships that could be construed as a potential conflict of interest.

## Footnotes

^1^Digit ratio reflects fetal testosterone relative to estradiol exposure, with lower digit ratios reflecting higher testosterone/lower estradiol exposure (Lutchmaya et al., 2004). For readability, we heretofore refer to testosterone exposure.

^2^Participants also completed the Perspective Taking Scale (Davis, 1983; de Corte, 2007; *M* = 2.97, SD = 0.43; relationship to Empathic Concern, *r* = 0.29, *p* < 0.01) and we explored its effects, alone or in interaction with Treatment and 2D:4D. Including or excluding Perspective Taking had no effects whatsoever.
